# Acute abdomen caused by a hernia of the broad ligament: a case report and literature review

**DOI:** 10.1097/MS9.0000000000005758

**Published:** 2026-10-19

**Authors:** Dovilė Kielaitė, Rusnė Gutauskaitė, Mindaugas Šilkūnas

**Affiliations:** a Faculty of Medicine, Vilnius University, Vilnius, Lithuania; b Clinic of Obstetrics and Gynaecology, Faculty of Medicine, Institute of Clinical Medicine, Vilnius University, Vilnius, Lithuania

**Keywords:** acute abdomen, bowel ischemia, broad ligament hernia, case report, gynecological emergency

## Abstract

Broad ligament hernia is a rare cause of internal herniation and an acute abdomen in women, accounting for less than 1% of gynecological emergencies. Due to its nonspecific presentation and often inconclusive imaging findings, diagnosis is frequently delayed, increasing the risk of bowel ischemia and necrosis. We present a case of small bowel herniation through a defect in the broad ligament in a female patient admitted with acute abdominal pain. Initial contrast-enhanced computed tomography did not demonstrate definitive signs of internal herniation, despite progressive clinical deterioration. Owing to worsening symptoms, surgical exploration was undertaken, which revealed extensive small bowel herniation with ischemia and necrosis, requiring bowel resection. A narrative literature review was performed to contextualize the case and assess current diagnostic and surgical approaches to broad ligament hernias. This case highlights the diagnostic challenge posed by this rare pathology and shows the limitations of imaging when clinical findings are discordant. A broad ligament hernia should be considered in the differential diagnosis of an acute abdomen in women, particularly when risk factors such as prior pelvic surgery or multiparity are present. Early surgical exploration is essential to prevent severe complications and improve patient outcomes.

## Introduction

Broad ligament hernia (BLH) is a rare type of internal hernia (IH), representing approximately 4–7% of all IH cases^[^1^]^. It involves abdominal organs, typically the small intestine, extending through a defect (either congenital or acquired) in the broad ligament (BL) of the uterus^[^2^]^. BL hernia accounts for less than 1% of cases of acute abdomen in gynecology^[^3^]^, making it a complex pathology for clinicians to unravel, since the differential diagnosis includes ectopic pregnancy, ovarian torsion, ruptured cysts, pelvic inflammatory disease, or appendicitis. Notably, BLH is the most common IH in the pelvis^[^4^]^.

HIGHLIGHTS

- Broad ligament hernia is a rare but life-threatening cause of acute abdomen, with a nonspecific presentation and low rates of preoperative diagnosis.
- This case demonstrates that contrast-enhanced CT may be inconclusive despite progressive clinical deterioration, highlighting the limitations of imaging in internal pelvic hernias.
- Recurrent emergency presentations with worsening pain preceded the diagnosis, which ultimately revealed extensive small-bowel herniation with ischemia requiring resection.
- Prior pelvic surgeries (cesarean sections) likely contributed to the development of the broad ligament defect, consistent with reported risk factors.
- Early surgical exploration should be considered when clinical findings are discordant with imaging to prevent bowel necrosis and reduce morbidity.

The BL of the uterus is a double-layered fold of the peritoneum that attaches the uterus to the lateral pelvic sidewalls^[^5^]^. Depending on whether one or both layers of the BL are involved, Hunt *et al* proposed a two-type classification: the fenestra type, involving both layers, and the pouch type, in which only a single peritoneal layer is disrupted^[^1^]^. Cilley suggested another – anatomical – classification of BLHs, which describes three types of pathology: type I (caudal to the round ligament), type II (above the round ligament, including the suspensory ligament of the ovary, the mesosalpinx, or the mesovarium), and type III (on the two-layered surface of the round ligament and mesometrium)^[^2^]^ (Fig. 1).

**Figure 1.**
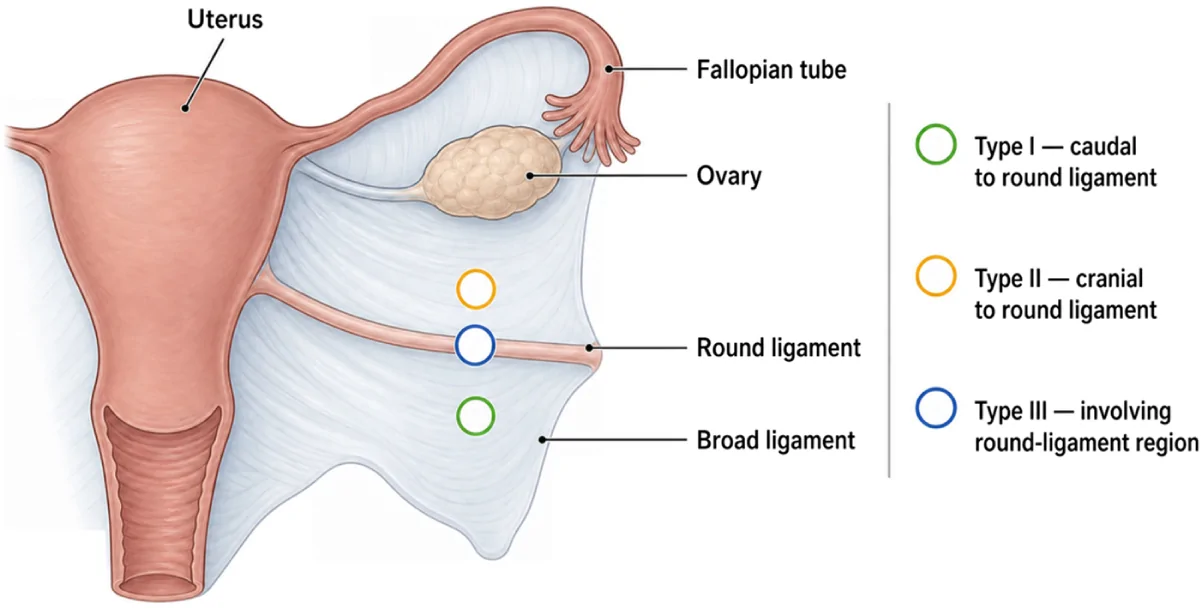
Original schematic representation of the Cilley anatomical classification of broad-ligament defects: type I, caudal to the round ligament; type II, cranial to the round ligament; and type III, involving the round-ligament region.

The common etiologies of IHs are adhesions after surgery, inflammatory bowel disease, and malignancy^[^2^]^. As bariatric surgeries, such as Roux-en-Y gastric bypass, become more common, the incidence of IHs may rise ^[^2^]^.

Predisposing risk factors described in the literature often include multiparity, a history of abdominal or pelvic surgery, pelvic inflammatory disease or endometriosis, obstetric trauma from childbirth, and congenital anomalies of the BL, especially in patients without prior surgery or trauma^[^2^]^. In addition, Allen-Masters syndrome is a clinical manifestation of herniation through the BL, the cul-de-sac, or the uterosacral ligament^[^1^]^. Usually, these ligaments are weakened by traumatic obstetric tears or chronic endometriosis^[^1^]^.

The clinical presentation of BLH is typically nonspecific, with a wide range of symptoms, most often involving acute abdominal pain, nausea, and vomiting^[^1,2^]^, which significantly complicates timely diagnosis. However, early recognition is essential, as delayed management is associated with an increased risk of bowel ischemia and potentially fatal outcomes.

We present a case highlighting the importance of considering uncommon causes of acute abdominal pain and demonstrating the complexity of the diagnostic process.

## Materials and methods

This case report was prepared in accordance with the CARE guidelines, and clinical information was collected retrospectively from the electronic medical record. A focused narrative literature review was conducted in PubMed, Scopus, and Web of Science using combinations of the terms “broad ligament hernia,” “broad ligament defect,” “internal hernia,” “small bowel obstruction,” “acute abdomen,” “bowel ischemia,” “pelvic hernia,” and “case report.” Reference lists of relevant articles were also examined to identify additional reports. Eligible publications were English-language case reports, case series, reviews, or imaging studies that described the diagnosis, imaging findings, operative management, or outcomes of uterine BLH. Publications concerning other types of internal or pelvic hernia, reports without clinically relevant information on BLH, conference abstracts without sufficient case details, and duplicate reports were excluded. No publication-year restriction was applied to the broader narrative review because of the rarity of the condition. The 31 included publications were accessed through database-linked journal pages, PubMed Central, or institutional full-text access and were used to inform the background and discussion. For the comparative table, we selected 21 individual cases published from 2020 onward that provided sufficient patient-level data on imaging, operative findings, bowel viability, and management. Extracted variables included age, clinical presentation, relevant obstetric and surgical history, imaging findings, intraoperative findings, bowel viability, defect location, and surgical management. Findings were synthesized descriptively; no meta-analysis or formal assessment of study quality was performed. Because this was a focused narrative review rather than a systematic review, PRISMA methods and a PRISMA flow diagram were not applied. ChatGPT (OpenAI, San Francisco, CA, USA) was used to assist with English-language editing of the Abstract, Introduction, Case Presentation, Discussion, and Conclusions. It was not used to select publications, extract data, interpret clinical findings, perform statistical analysis, or generate references. It was also used to create illustrations. All AI-assisted text was critically reviewed, corrected, and approved by the authors, who take full responsibility for the manuscript’s content.

## Case presentation

A 45-year-old woman presented to the Emergency Department with a 6-hour history of acute, severe right-sided abdominal pain (visual analog scale [VAS] score: 6–8/10), nausea, and vomiting. The symptoms had begun during the night with no identifiable precipitating event. She reported no urinary symptoms or change in bowel function. Her medical history included hypothyroidism and two cesarean deliveries more than 10 years earlier. She had regular menstrual cycles, and her last menstrual period had begun 4 days before presentation.

Upon examination, the patient was afebrile, with stable vital signs (heart rate 72 beats per minute, blood pressure 127/87 mmHg). Physical assessment revealed severe pain throughout the abdomen; the abdomen was soft; peritoneal signs were negative; and at the time, acute appendicitis was not suspected. Rectal examination was unremarkable. The patient initially refused an internal gynecologic examination and vaginal sonography because of severe pain and discomfort.

The abdominal ultrasound scan revealed free fluid between the intestinal loops and in the left iliac fossa. Abdominal radiography did not reveal free air in the abdominal cavity suggestive of perforated ulcers or signs of obstruction. Contrast-enhanced computed tomography (CECT) of the abdomen and pelvis confirmed free fluid in the pelvis; no other specific findings were mentioned. Information regarding the radiologist’s subspecialty and the availability of multiplanar reconstructions could not be retrieved retrospectively. The initial report did not describe a closed bowel loop, pelvic transition points, bowel dilatation, or displacement of the uterus or adnexa. Laboratory tests showed normocytic, normochromic anemia (RBC 4.09 × 10^12^/L, MCV 86.6 fL, MCH 29.4 pg, MCHC 340 g/L) and hyperglycemia (serum glucose 7.07 mmol/L). At that time, the patient’s pain improved following intravenous administration of a balanced isotonic crystalloid solution (Sterofundin ISO®, 100 mL), metamizole sodium 2 g, drotaverine 80 mg, metoclopramide 10 mg (Elitan®), tramadol 100 mg, and esomeprazole 40 mg. The patient was hemodynamically stable, the abdomen was soft without peritoneal signs, abdominal radiography showed no obstruction or free intraperitoneal air, and CECT showed only nonspecific pelvic free fluid. Because the pain improved after treatment and no condition requiring emergency surgery was identified, the patient was discharged with instructions to return immediately if the pain persisted or worsened.

The patient returned 4 hours later because of worsening abdominal pain. Repeated clinical examinations remained inconclusive, and no new objective abnormality requiring emergency intervention was documented. After analgesic treatment with 50 mg of pethidine hydrochloride administered intramuscularly and intravenous administration of 1000 mL of 0.9% sodium chloride, the pain improved, and she was discharged again. However, the pain recurred the following morning, prompting a third presentation to the Emergency Department.

On presentation, the patient exhibited elevated blood pressure (145/80 mmHg) and tachycardia (98 beats per minute). The woman was restless and assumed a forced position. The pain intensity was rated at 9/10, and the abdomen was tense on palpation. Gynecological examination revealed bloody vaginal discharge consistent with menstruation, with no other abnormal features.

Ultrasonography again showed free pelvic fluid, with a maximum pocket depth of 3 cm, but no definite cause was identified. Laboratory testing demonstrated leukocytosis (WBC 14.71 × 10^9^/L), an increase in serum glucose to 9.40 mmol/L, and an increase in CRP from 0.58 mg/L on day 1 to 5.75 mg/L on day 2. Because of recurrent presentations and progressive abdominal pain, the patient was admitted to the Department of Abdominal Surgery. Emergency diagnostic laparoscopy identified an IH containing necrotic small bowel through a defect in the left broad-ligament (Fig. 2A). The operation was converted to a midline laparotomy, and approximately 100 cm of nonviable ileum was resected, with restoration of bowel continuity (Fig. 2B). No intraoperative complications occurred. Recovery was uneventful. On postoperative day 8, the WBC had decreased to 7.04 × 10^9^/L and CRP was 15.04 mg/L. The patient was clinically stable and was discharged with planned follow-up.

**Figure 2.**
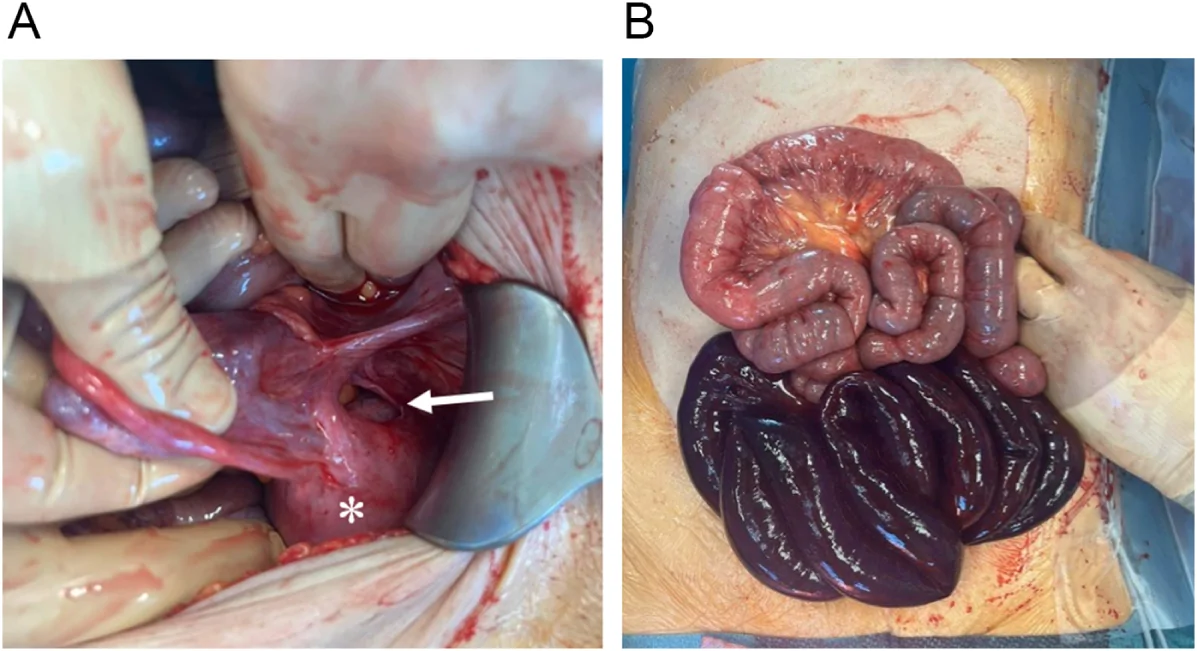
Intraoperative findings: (A) Type I defect in the broad ligament (arrow; * denotes the uterus); (B) Necrotic small intestine (*).

## Discussion

The principal contribution of this case is not the demonstration that broad-ligament hernia is rare or diagnostically difficult, as these features are already well established. Rather, the case documents rapid progression from nonspecific symptoms and isolated pelvic free fluid to extensive bowel necrosis over three Emergency Department presentations. It illustrates the limitations of relying on temporary analgesic response and a single early nondiagnostic CECT examination when severe symptoms recur. An approximately 100 cm ileal resection further demonstrates the potential consequences of delayed recognition, even though similarly extensive resection has occasionally been reported before.

In retrospect, the second presentation represented an important opportunity for prolonged observation, repeated surgical assessment, or reconsideration of the initial imaging findings. Although temporary improvement with analgesia and the absence of peritoneal signs reduced the apparent urgency, recurrent severe pain after an earlier nondiagnostic CECT examination should have raised concern for an evolving intra-abdominal process. This case, therefore, illustrates that symptomatic improvement with analgesia does not exclude intermittent bowel entrapment or progressive strangulation.

In women of reproductive-age presenting with pelvic pain, diagnostic reasoning is frequently directed toward gynecological pathology. Based on the clinical, laboratory, and imaging findings, the differential diagnosis should include perforated acute appendicitis, generalized small-bowel obstruction with possible strangulation, and acute gynecological emergencies^[^6^]^. The latter encompasses ovarian torsion, ovarian cyst hemorrhage or rupture, pelvic inflammatory disease with formation of a tubo-ovarian abscess, ruptured ectopic pregnancy, and acute complications of endometriosis^[^7^]^. Typically, all these pathologies exhibit characteristic radiologic findings, which were not the case in the present case. Preoperative diagnosis of broad-ligament hernia (BLH) might have been facilitated if imaging had demonstrated signs of small-bowel obstruction. However, CECT revealed only free intrapelvic fluid measuring 3 cm, as ultrasound (US) examination later confirmed. Although early reports emphasized the limitations of CT in diagnosing broad-ligament hernia^[^8^]^ multidetector CT and greater awareness of characteristic pelvic anatomical relationships have improved preoperative recognition in more recent reports. Suggestive findings include a closed-loop small-bowel obstruction in the pelvis, proximal bowel dilatation, two adjacent transition points lateral to the uterus, widening of the distance between the uterus and ovary, displacement of the uterus or adnexa, and associated pelvic free fluid^[^9,10^]^. Nevertheless, these findings may be subtle or absent during the early stage of herniation. In the present case, CT was reported to show only pelvic free fluid, without bowel dilatation, a closed loop, identifiable transition points, or characteristic displacement of the uterus or adnexa. The examination may therefore have been performed before these features became conspicuous. In addition, because BLH is rare and there were no clear signs of bowel obstruction, a broad-ligament defect was not initially suspected. The original CT images were not reinterpreted specifically for this report; consequently, it cannot be determined whether subtle signs were absent or were present but unrecognized. The principal concern was therefore the subsequent discordance between a nondiagnostic early examination and progressive clinical deterioration.

Intraoperative diagnosis of BLH fits within the general diagnostic course, given that the reported preoperative detection rate is only 36–46.4%^[^3,11^]^. In the presented case, diagnostic laparoscopy revealed a Cilley type I BL herniation, which is the most common anatomical variant reported in the literature^[^2^]^. The small intestine herniating through the defect was already necrotic, necessitating conversion to a longitudinal laparotomy. In the study by Byrne *et al*, transition from an initial laparoscopic approach to open surgery occurred in 38.6% of cases; nevertheless, no additional complications were observed after conversion compared with primary open surgery^[^12^]^. After resection of the necrotic intestine, bowel continuity can be restored either by primary anastomosis (as in our case) or by stoma formation^[^13^]^. The latter is reported more frequently in the literature^[^14^]^.

Importantly, the patient had a history of two cesarean deliveries, which is a known risk factor for BL fenestration – prior surgeries themselves could have weakened or damaged the structural integrity of the BL of the uterus by micro-trauma, surgical traction, or altered pelvic anatomy^[^2^]^. Similar to other pathologies, BL defects can be congenital or acquired. Abnormal development of the BL or cystic ruptures of remnants of the mesonephric or Müllerian ducts are possible primary causes^[^9^]^. The integrity of the ligament may be compromised over time by trauma from surgery, vaginal manipulation, or pregnancy^[^2,9^]^. Multiparous women are at an increased risk due to reduced BL elasticity^[^15^]^. Endometriosis can promote BL defects through mesenteric damage^[^1^]^, while pelvic inflammatory disease contributes via adhesion formation and anatomical distortion^[^16^]^. In addition, an association between bariatric surgery and the formation of IH has been reported^[^17^]^.

The present case demonstrates that the clinical and radiological manifestations of BLH may evolve over time. Initial imaging and laboratory findings were inconclusive, whereas progressive strangulation subsequently resulted in clinical deterioration and extensive bowel necrosis. Recent reports describe both successful CT-based preoperative diagnoses and cases in which BLH mimicked adnexal torsion or another pelvic disorder^[^8,11,18–20^]^. These contrasting observations support repeated clinical assessment and multidisciplinary review of imaging when symptoms progress despite an initially nondiagnostic examination.

Clinicians should consider an IH through a BL defect in women with an acute abdomen, especially those with prior pelvic surgery or multiparity, even in the absence of clear radiologic evidence of bowel obstruction. Moreover, early surgical exploration should be strongly considered when clinical deterioration is discordant with imaging findings.

Continuous accumulation and analysis of such reported cases may help refine diagnostic algorithms and imaging criteria for early recognition of this rare condition. It is important to note that the characteristics of the presented case – acute, intense abdominal pain accompanied by nausea and vomiting in the absence of an alternative diagnosis – should prompt suspicion of a bowel obstruction due to an IH. In such scenarios, particularly when radiologic findings are inconclusive, early diagnostic laparoscopy may facilitate timely diagnosis, thereby preventing bowel ischemia and necrosis.

After a literature review of 21 similar reports published in the past 5 years, we identified several similarities and differences between our case (Table 1).

**Table 1:** Clinical timeline from symptom onset to discharge.

Time point	Clinical findings	Investigations	Management/outcome
Initial presentation (6 h after onset)	Right-sided abdominal pain, VAS 6–8/10; nausea and vomiting; HR 72 beats/min; BP 127/87 mmHg; soft abdomen without peritoneal signs	US: pelvic/interloop free fluid; radiograph: no obstruction or free air; CECT: pelvic free fluid only; glucose 7.07 mmol/L	Symptomatic intravenous treatment; pain improved; discharged
Second presentation (4 h later)	Worsening abdominal pain; repeat examinations inconclusive	No new diagnostic finding documented	Pethidine and intravenous 0.9% sodium chloride; discharged after improvement
Third presentation (next morning)	Pain 9/10; restlessness; tense abdomen; HR 98 beats/min; BP 145/80 mmHg	US: pelvic free fluid, 3-cm pocket; WBC 14.71 × 10^9^/L; CRP increased from 0.58 to 5.75 mg/L	Admitted to abdominal surgery
Emergency operation	Progressive pain with discordant imaging findings	Laparoscopy: left type I BL defect with necrotic ileum	Conversion to midline laparotomy; approximately 100 cm of ileum resected; bowel continuity restored
Postoperative day 8	Clinically stable; uncomplicated recovery	WBC 7.04 × 10^9^/L; CRP 15.04 mg/L	Discharged with planned follow-up

Relevant predisposing factors were inconsistently reported in the included cases. Where such information was available, the most frequently described factors were previous abdominal or pelvic surgery – particularly cesarean delivery – and multiparity. Less commonly reported associations included endometriosis, pelvic inflammatory disease, and other previous pelvic procedures or obstetric trauma. Several cases occurred in women without a history of pelvic surgery or trauma, suggesting a possible congenital broad-ligament defect. These findings are consistent with the recent systematic review by Dowers *et al*^[^2^]^, which identified previous abdominal surgery and multiparity as the principal factors associated with broad-ligament hernia. However, because the source case reports did not consistently report risk-factor histories, their relative frequencies should be interpreted cautiously. First, it is notable that there is no specific age at which this pathology occurs; the youngest patient was 34 years old^[^20^]^ and the oldest – 88 years old^[^21^]^. Free fluid in the pelvic cavity was detected by radiologic imaging in 8 cases; in 2 of them, it was the only visualized feature, as it was in the present situation. By contrast, more pathological findings are usually identified, the most common being small-bowel obstruction and dilatation of the intestines. BL herniation was identified or suspected in 3 clinical situations; in 7 cases, some abnormalities of uterine location or in the adnexal region were found; thereby, in these situations, the diagnosis could be confirmed preoperatively. The most common surgical treatment was laparoscopy (9 cases, plus one case in which a minor open surgical approach was required in addition to laparoscopy^[^22^]^); less frequently (7 cases), open surgery was the primary intervention, and only 4 cases were managed as in ours – diagnostic laparoscopy converted to laparotomy. Ischemia of some part of the herniated intestine occurred in fewer than half (8 cases) of the cases. The length of the necrotic segment was often significantly shorter (ranging from 10 cm to 40 cm) than in our case (100 cm), and such significant bowel removal was necessary in only one case^[^23^]^. After resection of the necrotic segment, intestinal integrity was restored by performing an end-to-end anastomosis in most scenarios (3 cases); other authors reported choosing side-to-side (1 case) and primary anastomosis (1 case). The defect of the BL of the uterus was detected more often on the left side (13 cases) than on the right side (7 cases), and in one presentation both sides of the ligament were damaged. The defect was sutured using various techniques, described in Table 2.

**Table 2:** Characteristics, imaging findings, and operative management of 21 recently reported broad ligament hernia cases.

Reference	Age, years	Imaging findings	Surgical approach	Bowel ischemia	Side of BLD	Bowel and defect management
^[^3^]^	39	Partially displayed bilateral adnexal area, free fluid, SBD (US), air-fluid levels in the left lower abdomen (CT)	LS	No	L	BLD – 3-0 absorbable suture
^[^6^]^	47	Free fluid (US)	C	Yes	L	RBI – end-to-end anastomosis
^[^10^]^	35	Air-fluid levels (X-ray), free fluid, SBD (CECT)	LS	No	L	BLD – marsupialization, additional: salpingectomy
^[^10^]^	42	Air-fluid levels (X-ray), SBD, possible BL herniation (CECT)	LS	No	L	BLD – suture (n. m. specifics)
^[^11^]^	46	Free fluid (CT), SBD, UT compression, left ovarian venous dilatation and tortuousness (CECT)	LS	No	L	n. m.
^[^16^]^	49	Tortuous left tubular mass in the pelvic cavity behind the UT (CT)	LS	Yes	L	BLD – continuous sutures
^[^17^]^	42	SBO, SB located in the left lower abdomen (CT)	LS	No	L	BLD – titanium surgical clips
^[^24^]^	34	Free fluid (US, CT)	OS	No	R	n. m.
^[^25^]^	71	SBD (X-ray, CT), closed-loop invaginated into the BL, UT and rectosigmoideum colon displacement (CT)	LS	No	L	BLD – ultrasonic coagulation
^[^18^]^	73	SBD (X-ray, CT), closed-loop obstruction, free fluid, UT displacement (CT, CECT)	OS	No	L (and R)	BLD – non-absorbable sutures
^[^26^]^	75	SBO, SBD (CECT)	LS	No	R	BLD – nonabsorbable V-Lock® 2.0 suture, superficial peritoneal stitches
^[^19^]^	57	SBD (X-ray), possible SB internal herniation through BL (CT)	OS	No	R	BLD – absorbable sutures
^[^20^]^	30	Closed-loop SBD in the pelvis (CT)	OS	No	R	BLD – continuous sutures, additional: retrograde salpingectomy
^[^27^]^	62	SBO, free fluid (CT)	C	Yes	L	RBI – end-to-end anastomosis
^[^28^]^	70	SBD, SB wall thickening (CECT)	C	Yes	L	n. m., additional: left salpingoophorectomy
^[^22^]^	57	SBD next to the UT (CECT)	LS + OS	Yes	L	RBI – end-to-end anastomosis
^[^23^]^	42	SBD, air-fluid levels (X-ray), free fluid, ileum wall thickening (CECT)	OS	Yes	L	RBI – side-to-side isoperistaltic anastomosis, BLD—continuous polydioxanone 2-0 suture
^[^21^]^	88	SBD (X-ray, CECT), free fluid (CECT)	OS	Yes	R	RBI – adhesiolysis, BLD—polydioxanone 2-0 continuous sutures
^[^29^]^	48	SBO (X-ray)	OS	Yes	L	RBI – primary anastomosis, BLD – interrupted sutures
^[^30^]^	52	Closed-loop SBO, UT dislocation (CT)	C	No/yes	R	BLD – continuous suture, additional: right salpingectomy
^[^31^]^	34	SBD, enlarged distance between the UT and the ovary (CT)	LS	No	R	BLD – 2/0 Vicryl® sutures

BL, broad ligament; BLD, broad ligament defect; C, conversion; CECT, contrast-enhanced computed tomography; CT, computed tomography; L, left; LS, laparoscopy; n.m., not mentioned; OS, open surgery; R, right; RBI, restoration of bowel integrity; SB, small bowel; SBD, small bowel dilatation; SBO, small bowel obstruction; US, ultrasound; UT, uterus.

## Conclusions

BLH is a rare but clinically significant pathology that can lead to severe complications if diagnosis is delayed. It accounts for less than 1% of gynecological causes of acute abdomen, which contributes to diagnostic difficulty and frequent initial misdiagnosis. BL herniation has been reported across a wide age range, and free pelvic fluid is commonly described as an associated imaging finding. Surgical management most frequently involves laparoscopy, although open surgery or conversion from diagnostic laparoscopy to laparotomy may be required depending on intraoperative findings and bowel viability. The presented case highlights the diagnostic challenge of this condition, as the patient’s clinical status deteriorated despite initially inconclusive computed tomography findings, resulting in delayed surgical intervention and extensive bowel necrosis. This highlights the limitations of imaging and the importance of clinical judgment. Clinicians should maintain a high index of suspicion for BLH in women presenting with an acute abdomen, particularly in the presence of risk factors such as prior pelvic surgery or multiparity. Early surgical exploration should be considered when clinical deterioration is discordant with imaging findings to prevent bowel ischemia and associated morbidity.

## Limitations

This narrative review has several limitations. It was not designed as a systematic review; no formal risk-of-bias assessment was undertaken; and the included case reports varied substantially in the completeness of clinical history and outcome reporting. The numerical summaries should, therefore, be interpreted as descriptive observations from published cases rather than estimates of incidence, diagnostic accuracy, or comparative treatment effectiveness.

## Ethical approval

Ethical approval for this case report was granted on 20 February 2026 (approval No. SR-1276).

## Consent

Written informed consent was obtained from the patient for publication of this case report and any accompanying images.

## Sources of funding

This research received no external funding.

## Author contributions

D.K. and R.G.: Conceptualization, methodology, resources, formal analysis, visualization, writing, writing – original draft. M.S.: Writing – review and editing.

## Conflicts of interest disclosure

The authors declare no conflict of interest.

## Research registration unique identifying number (UIN)

Not applicable, as this is a case report and not a clinical trial or first-in-human study.

## Guarantor

Mindaugas Šilkūnas.

## Provenance and peer review

Not commissioned; externally peer reviewed.

## Data Availability

No new datasets were generated or analyzed in this study. The clinical information relevant to this case is included in the article. Additional patient-level data are not publicly available because of privacy and confidentiality restrictions.

## References

[1] Sajan A Hakmi H Griepp DW. Herniation through defects in the broad ligament. JSLS 2021;25:e202000112. 10.4293/JSLS.2020.00112 PMC8241289 34248336

[2] Dowers K Costantini S Montalbano MJ. Characteristics and associated risk factors of broad ligament hernia: a systematic review. Med Sci Monit 2025;31:e946710. 10.12659/MSM.946710 PMC11773988 39849826

[3] He J Wen Y Zhang B. Broad ligament defect causing internal hernia: a case report and literature review. Medicine (Baltimore) 2025;104:e45264. 10.1097/MD.0000000000045264 PMC12558271 41137286

[4] Monica ML Antonella M Gloria A. Internal hernias: a difficult diagnostic challenge. Review of CT signs and clinical findings. Acta Biomed 2019;90:20–37. 10.23750/abm.v90i5-S.8344 PMC6625567 31085971

[5] Craig ME Sudanagunta S Billow M. Anatomy, abdomen and pelvis: broad ligaments. In: StatPearls. StatPearls Publishing; 2025. Accessed. 4 December 2025. http://www.ncbi.nlm.nih.gov/books/NBK499943/. 29763118

[6] Sathiyanathan K Tharmakulasingham N Dias S. When gynecological anatomy becomes a surgical emergency: a rare internal hernia case. Cureus 2025;17:e87766. 10.7759/cureus.87766 PMC12337064 40792316

[7] Franco PN García-Baizán A Aymerich M. Gynaecological causes of acute pelvic pain: common and not-so-common imaging findings. Life 2023;13:2025. 10.3390/life13102025 PMC10608316 37895407

[8] Suzuki M Takashima T Funaki H. Radiologic imaging of herniation of the small bowel through a defect in the broad ligament. Gastrointest Radiol 1986;11:102–04. 10.1007/BF02035043 3943668

[9] Matsunami M Kusanagi H Hayashi K. Broad ligament hernia successfully treated by laparoscopy: case report and review of literature. Asian J Endosc Surg 2014;7:327–29. 10.1111/ases.12119 25354380

[10] Rohatgi Y Harvitkar RU Raut V. Broad ligament hernia: two contrasting ways to a common goal - Two case reports with review of literature. Int J Surg Case Rep 2021;85:106198. 10.1016/j.ijscr.2021.106198 PMC8327646 34304087

[11] Kubota W Sakuma A Katada R. Usefulness of early diagnosis of small bowel obstruction due to broad ligament hernia using multidetector computed tomography: a case report. J Surg Case Rep 2022;2022:rjab598. 10.1093/jscr/rjab598 PMC8759510 35047179

[12] Byrne J Saleh F Ambrosini L. Laparoscopic versus open surgical management of adhesive small bowel obstruction: a comparison of outcomes. Surg Endosc 2015;29:2525–32. 10.1007/s00464-014-4015-7 25480627

[13] Schick MA Kashyap S Collier SA. Small bowel obstruction. In: StatPearls. StatPearls Publishing; 2025. Accessed. 26 January 2026. http://www.ncbi.nlm.nih.gov/books/NBK448079/. 28846346

[14] Kryvoruchko IA Boyko VV Sartelli M. Surgical treatment of acute small bowel obstruction: clinical and laboratory parameters associated with strangulation and early mortality after surgery. Wiad Lek 2022;75:2891–900. 10.36740/WLek202212101 36723300

[15] Ahuja A Rengan SS Dey A. An unusual case of small-bowel obstruction: broad ligament hernia. J Minim Access Surg 2025;21:315–17. 10.4103/jmas.jmas_280_23 PMC12327788 39388357

[16] Shima K Nishida M Kai K. A case of uterine broad ligament hernia difficult to distinguish from adnexal torsion. Cureus 2025;17:e78790. 10.7759/cureus.78790 PMC11807731 39931504

[17] Kaleem A Othman D Yanez C. Acute internal pelvic hernia: a complex diagnostic challenge. CRSLS 2025;12:e202400074. 10.4293/CRSLS.2024.00074 PMC11938174 40151650

[18] Kaneko K Takano M Fukasawa T. Preoperative Diagnosis and Indocyanine Green (ICG) fluorescence-guided surgery for uterine broad ligament hernia: a case report. Cureus 2025;17:e80867. 10.7759/cureus.80867 PMC12008819 40255816

[19] Moses Wong YK Teng WW Sharon Chong ZC. Holes can be perilous: a rare presentation of intestinal obstruction - Herniation through the broad ligament. Radiol Case Rep 2024;19:1309–12. 10.1016/j.radcr.2023.12.067 PMC10825535 38292799

[20] Slimi Y Frikal M Mahmoudi M. Small bowel obstruction unmasking an internal hernia through a broad ligament defect: a case report. J Surg Case Rep 2025;2025:rjaf074. 10.1093/jscr/rjaf074 PMC11844244 39989726

[21] Anagha TR Hassan N Manikanta KS. Internal hernia through acquired broad ligament defect: a case report. Cureus 2024;16:e66766. 10.7759/cureus.66766 PMC11391922 39268311

[22] Song JH Kim JS. Strangulated internal hernia through a defect of the broad ligament: a case report of laparoscopic surgery. Ann Coloproctol 2023;40:S44–7. 10.3393/ac.2022.00906.0129 PMC11162844 37102211

[23] Karthik N Lodha M Banerjee N. Quain hernia masquerading as mesenteric ischemia. Cureus 2024;16:e61664. 10.7759/cureus.61664 PMC11223663 38966483

[24] MacDonald S Stokes E Sharaf U. Quain hernia - A rare cause of acute small bowel obstruction. A case report and an updated literature review. Int J Surg Case Rep 2021;89:106599. 10.1016/j.ijscr.2021.106599 PMC8605219 34794072

[25] Sugishita T Kato S Ishikawa A. Less invasive treatment for broad ligament hernia: a case report. Int J Surg Case Rep 2020;73:172–75. 10.1016/j.ijscr.2020.07.012 PMC7369453 32693229

[26] Mohamed L Mougib H Madani AM. Acute intestinal obstruction revealing an internal hernia of the broad ligament following promontofixation: a case report. Cureus 2025;17:e90080. 10.7759/cureus.90080 PMC12433254 40951157

[27] Arif SH Mohammed AA. Strangulated small-bowel internal hernia through a defect in the broad ligament of the uterus presenting as acute intestinal obstruction: a case report. Case Rep Womens Health 2021;30:e00310. 10.1016/j.crwh.2021.e00310 PMC8040108 33868965

[28] Reyes N Smith LE Bruce D. Strangulated internal hernia due to defect in broad ligament: a case report. J Surg Case Rep 2020;2020:rjaa487. 10.1093/jscr/rjaa487 PMC7700802 33294168

[29] Nandi P Jain S Kainth A. Internal hernia through a congenital defect in broad ligament: a rare and elusive cause of intestinal obstruction. Cureus 2022;14:e24769. 10.7759/cureus.24769 PMC9167437 35676990

[30] Takahashi M Yoshimitsu M Yano T. Rare contents of an internal hernia through a defect of the broad ligament of the uterus. Case Rep Surg 2021;2021:5535162. 10.1155/2021/5535162 PMC8181115 34194864

[31] Bouzid A Ameur HB Fourati K. Laparoscopic repair of the broad ligament hernia: a case report. Int J Surg Case Rep 2023;106:108160. 10.1016/j.ijscr.2023.108160 PMC10123247 37058797

